# Supplementary material for: ProteinShader: illustrative rendering of macromolecules
Source: BMC Struct Biol. 2009 Mar 30;9:19. doi: 10.1186/1472-6807-9-19 (PMC2672931; doi:10.1186/1472-6807-9-19)
Supplement: Additional file 1 — ProteinShader program without source code. This compressed file contains the complete ProteinShader program including associated libraries, but no source code. A README.txt file gives an overview of the ProteinShader distribution, and the index.html file in the help subdirectory has directions on getting started with the program as well as a set of tutorials. [file 1472-6807-9-19-S1.zip › ProteinShader-beta-0_9_4-binary/help/tutorials/1A05.html]

ProteinShader Tutorial: 1A05


# ProteinShader Tutorial


♦ Home


♦ Download


♦ Getting Started

Choose a Link:

Installation

Running

Troubleshooting

PDB Files


♦ Tutorials

Choose a Link:

List of Proteins

Style Options

Saving Images


♦ General Controls

Choose a Link:

Menu Bar

Keyboard Shortcuts

Mouse Motion

Motion Panel

Antialiasing


♦ Cartoon Controls

Choose a Link:

Control Panels

Selection Panel

Radio Buttons

Decorations Panel

Adding Textures

Color Panel

Visibility Panel

Side Chains Panel


♦ Atom Controls

Choose a Link:

Control Panels

Selection Panel

Radio Buttons

Color Panel

Visibility Panel

Scale Panel

Tiling Panel


♦ Developers

Choose a Link:

Compiling

Running

Code Docs

Developer Links


## 3-Isopropylmalate Dehydrogenase: Three-Layer (Alpha-Beta-Alpha) Sandwich [PDB:1A05]

The 3-isopropylmalate dehydrogenase enzyme catalyzes dehydrogenation
and decarboxylation reactions that are important in the biosynthetic pathway
for the amino acid leucine [1]. The structure of this
bifunctional dimeric enzyme from *Thiobacillus ferrooxidans* has been
solved by x-ray crystallography
[1, 2],
and can be loaded into the ProteinShader program by selecting Open from the
File menu and then using the
file chooser box to select the 1A05.pdb file.


## Cartoon-style displays with halftoning

When the enzyme is first loaded, it will be displayed as a pen-and-ink style
drawing of ribbons and tubes. To obtain the orientation shown in Figure 1A,
use the
Orientation menu
above the canvas to select Bottom. It may also be helpful to zoom in a little
by using the middle mouse button or scroll-wheel (see
mouse motion control
for a summary of the mouse controls). Alternatively, if the canvas
has the focus (because it was clicked on), pressing the "i" key once
will zoom the camera in by 5 angstroms, while the "o" key will zoom the
camera out by 5 ansgstroms.

By default, alpha-helices are shown as tubes, while beta-strands are shown as
wide ribbons, and general loop regions are shown as thin ribbons. To place
more emphasis on the beta-strand ribbons, click on the β-Strands radio
button of the
Decorations subpanel,
and then select Vertical Bars from the Halftone Texture menu and None from the
BendTexture menu. To de-emphasize the loop regions, switch from the
Decorations subpanel
to the
Cartoon Visibility subpanel
and click on the Loops radio button before pressing the Translucent button.
The degree of translucency can be adjusted with the slider control below
the button.

With the loop regions faded out and the orientation as in Figure 1B, it should
be more obvious that the protein has two pockets, one above and a little
to the right of the large central domain, and one below and a little to the
left. Each pocket serves as a binding site for the enzyme's substrate,
3-isopropylmalate. By default, the ProteinShader program does not show
heterogens when a new structure is loaded, so go the
Visibility menu
above the canvas and select the Heterogens checkbox. The 3-isopropylmalate
molecules (one in each pocket) should appear as in Figure 1C, with spheres
colored according to atom type (gray for carbon and red for oxygen). A small
green sphere that is mostly obscured by the 3-isopropymalate is a magnesium ion.

The Visiblity menu above the canvas is intended as a quick and convenient way
to show or hide all amino acids, heterogens, or waters. For more fine grained
control, there is a
Cartoon Visibility subpanel
(for tube and ribbon segments) and an
Atom Visibility subpanel
(for space filling, balls and sticks, and sticks style displays). In a
cartoon-style display, heterogens are currently shown only as space filling
models, so manipulating the visibility of individual heterogens requires the
use of the
Atom Visibility subpanel.
For example, to get a better look at the magnesium ions while still
in the Figure 1C view, make sure that the Chain menu near the top left of the
Control Panel is showing chain A, and then select Heterogens from the menu
right below the Chain menu. The list of residues right below the menus should
now show four heterogens: two magnesium ions and two isopropylmalates.

To select the two isopropylmalates, click on HET\_IPM 401 and, while holding
down the keyboard Ctrl button, click on HET\_IPM 402. Now use the menu at the
top right of the Control Panel to change from the
Decorations subpanel
to the
Atom Visibility subpanel.
Click on the Selected radio button near the top of
the subpanel and make sure that Residues is showing in the menu below the radio
button. Click the Invisible button to hide the isopropylmalates. The two
magnesium ions should now be easy to see. Click the Opaque button to set the
isopropylmalates visible again.


|  |  |  |
| --- | --- | --- |
| **A** | **B** | **C** |

**Figure 1.** Ribbons and tubes style display of the enzyme 3-isopropylmalate
dehydrogenase. (A) Pen-and-ink style rendering with the default settings
for halftoning. (B) Same as part A after adding a vertical bars pattern to
the beta-strand ribbons and fading out the loop regions. (C) Same as part B,
but two molecules of the 3-isopropylmalate substrate are shown in their binding
sites.
The images were captured as described in the
Saving Images
section of the tutorials main page.


## Cartoon-style displays in color

The three-layer alpha-beta-alpha sandwich structure of 3-isopropylmalate
dehydrogenase may be a little easier to resolve using color as shown in Figure
2. Starting from the image shown in Figure 1C, go back to the
Decorations subpanel
and, after choosing Model from the menu under the Selected radio button,
click on the Plain button at the very bottom of the subpanel. In this context,
plain means color images with no special patterns mapped onto the surfaces of
the ribbons and tubes. By default, the beta-strands should be green, the
alpha-helices red, and the loop regions gray.

To emphasize the beta-strands, click on the β-Strands radio button to
select all beta-strands. Then use the Patterns button to apply Vertical Bars to
the beta-strands. The image should now appear as in Figure 2A. To rotate the
enzyme about its current vertical axis, hold down the shift key and right-click
while dragging the mouse horizontally across the canvas. Dragging about
halfway across the canvas should produce a rotation of about 180 degrees, which
should be close to the view shown in Figure 2B. Simply selecting Top from the
Orientation menu
will produce exactly the same orientation as in Figure 2B, but slowly rotating
the protein around will studying the secondary structure will give a better
sense of why the protein is referred to as an alpha-beta-alpha sandwich.


|  |  |
| --- | --- |
| **A** | **B** |

**Figure 2.** The three-layer alpha-beta-alpha structure of 3-isopropylmalate
dehydrogenase with alpha-helices in red and beta-strands in green.
(A) The view with Bottom selected from the
Orientation menu.
(B) Same as part A, except that the enzyme has been rotated 180 degrees by
selecting Top from the
Orientation menu.
The images were captured as described in the
Saving Images
section of the tutorials main page.


## Visualizing side chains in a binding site

The 3-isopropylmalate substrate binding site includes a hydrophobic pocket
that recognizes the substrate's gamma-isopropyl group
[1]. Visualizing the amino acid side chains
that form this pocket is a little easier if ribbons and tubes are shown as a
pen-and-ink style display, while the space filling side chains are shown in
color as in Figure 3. Starting from the image in Figure 2B, use the
Decorations subpanel
to select the entire Model from the menu under the Selected radio button,
and then apply Noise from the Halftone Texture menu and Diagonals 1 from the
Bend Texture menu. Next, click on the β-Strands radio button to select
all beta-strands, and apply Vertical Bars from the Halftone Texture menu and
None from the Bend Texture menu. The ribbons and tubes should now look similar
to Figure 3A, but to get a better view of the 3-isopropylmalate substrate
molecule in the lower half of figure, hold down the shift key and right-click
while dragging the mouse a short distance horizontally across the canvas.

To set space filling amino acid side chains visible in a ribbons and tubes style
display, use the menu at the top right of the Control Panel to switch to the
Cartoon Side Chains subpanel. Then go to the Chain menu near the top left
of the Control Panel and select chain B. With Amino Acids selected in the menu
right below the Chain menu, a list of all the amino acids in chain B should be
displayed. Scroll down the list and select VAL 193. Then make sure that
Residues is showing in the menu below the Selected radio button before clicking
on the Space Filling button of the
Cartoon Side Chains subpanel. By default, the spheres are colored by atom
type, which will be gray for carbon. To color all side chains by amino acid
type, switch to the
Atom Color subpanel,
and then select the entire Model from the menu below the Selected radio button
before clicking on the Amino Acid button at the bottom of the subpanel. The
VAL 193 side chain near the gamma-isopropyl group of the substrate in the lower
half of Figure 3A should now be green.

To see the other amino acid side chains that form the hydrophobic pocket,
go back to the
Cartoon Side Chains subpanel and switch to chain A before selecting
GLU 88, LEU 91, and LEU 92 and clicking on the space filling button (multiple
residues can be selected at the same time by holding down the Ctrl key while
left-clicking on residues). For a close up view of these residues as in
Figure 3B, the mouse can be used to zoom in or translate the image up or down
or left to right as described in the
Mouse Motion Control section.
To see the equivalent side chains near the gamma-isopropyl group in the top
half of Figure 3A, repeat the above procedure with VAL 193 on chain A and
GLU 88, LEU 91, and LEU 92 on chain B (chains A and B of this dimeric protein
are identical).


|  |  |
| --- | --- |
| **A** | **B** |

**Figure 3.** Visualization of a hydrophobic pocket that recognizes an
isopropyl group.
(A) The 3-isopropylmalate dehydrogenase enzyme is shown as tubes and ribbons
using halftoning for a pen-and-ink style appearance, while two substrate
molecules and subset of the side chains that recognize them are shown as a
space filling style display. (B) A closeup view of the lower portion of part A.
The 3-isopropylmalate substrate is colored by atom type, while side chains are
colored by amino acid type (red for glutamate and green for leucine and valine).
One of the side chains (Val 193) belongs to chain B, while three others (Glu 88,
Leu 91, and Leu 92) belong to chain A. For Glu 88 (the only side chain in red),
it is a CH2 group that is part of the hydrophobic pocket in close proximity to
the gamma-isopropyl group of the 3-isopropylmalate substrate.
The images were captured as described in the
Saving Images
section of the tutorials main page.


## References

1.
Imada K, Inagaki K, Matsunami H, Kawaguchi H, Tanaka H, Tanaka N, Namba K:
**Structure of 3-isopropylmalate dehydrogenase in complex with
3-isopropylmalate at 2.0 A resolution: the role of Glu88 in the
unique substrate-recognition mechanism.**
*Structure* 1998, **6**: 971-982.

2.
**3-Isopropylmalate Dehydrogenase PDB entry 1A05**
[http://www.rcsb.org/pdb/explore/explore.do?structureId=1A05]


---

|  |  |  |  |
| --- | --- | --- | --- |
| Hosted by |  |  |  |

Webmaster: Joe Weber,
joe.weber AT alumni.duke.edu
  
Web site last updated: December 30th, 2008
  
© 2007–2008 Joseph R. Weber
